# Assessment of Jugular Venous Blood Flow Stasis and Thrombosis During Spaceflight

**DOI:** 10.1001/jamanetworkopen.2019.15011

**Published:** 2019-11-13

**Authors:** Karina Marshall-Goebel, Steven S. Laurie, Irina V. Alferova, Philippe Arbeille, Serena M. Auñón-Chancellor, Douglas J. Ebert, Stuart M. C. Lee, Brandon R. Macias, David S. Martin, James M. Pattarini, Robert Ploutz-Snyder, L. Christine Ribeiro, William J. Tarver, Scott A. Dulchavsky, Alan R. Hargens, Michael B. Stenger

**Affiliations:** 1 KBR, Houston, Texas; 2 Institute of Biomedical Problems of the Russian Academy of Sciences, Moscow, Russian Federation; 3 University Hospital Trousseau, Tours, France; 4 National Aeronautics and Space Administration, Johnson Space Center, Houston, Texas; 5 Applied Biostatistics Laboratory, School of Nursing, University of Michigan, Ann Arbor; 6 Department of Surgery, Henry Ford Hospital, Detroit, Michigan; 7 Department of Orthopaedic Surgery, UC San Diego Medical Center, University of California, San Diego

## Abstract

**Importance:**

Exposure to a weightless environment during spaceflight results in a chronic headward blood and tissue fluid shift compared with the upright posture on Earth, with unknown consequences to cerebral venous outflow.

**Objectives:**

To assess internal jugular vein (IJV) flow and morphology during spaceflight and to investigate if lower body negative pressure is associated with reversing the headward fluid shift experienced during spaceflight.

**Design, Setting, and Participants:**

This prospective cohort study included 11 International Space Station crew members participating in long-duration spaceflight missions . Internal jugular vein measurements from before launch and approximately 40 days after landing were acquired in 3 positions: seated, supine, and 15° head-down tilt. In-flight IJV measurements were acquired at approximately 50 days and 150 days into spaceflight during normal spaceflight conditions as well as during use of lower body negative pressure. Data were analyzed in June 2019.

**Exposures:**

Posture changes on Earth, spaceflight, and lower body negative pressure.

**Main Outcomes and Measures:**

Ultrasonographic assessments of IJV cross-sectional area, pressure, blood flow, and thrombus formation.

**Results:**

The 11 healthy crew members included in the study (mean [SD] age, 46.9 [6.3] years, 9 [82%] men) spent a mean (SD) of 210 (76) days in space. Mean IJV area increased from 9.8 (95% CI, −1.2 to 20.7) mm^2^ in the preflight seated position to 70.3 (95% CI, 59.3-81.2) mm^2^ during spaceflight (*P* < .001). Mean IJV pressure increased from the preflight seated position measurement of 5.1 (95% CI, 2.5-7.8) mm Hg to 21.1 (95% CI, 18.5-23.7) mm Hg during spaceflight (*P* < .001). Furthermore, stagnant or reverse flow in the IJV was observed in 6 crew members (55%) on approximate flight day 50. Notably, 1 crew member was found to have an occlusive IJV thrombus, and a potential partial IJV thrombus was identified in another crew member retrospectively. Lower body negative pressure was associated with improved blood flow in 10 of 17 sessions (59%) during spaceflight.

**Conclusions and Relevance:**

This cohort study found stagnant and retrograde blood flow associated with spaceflight in the IJVs of astronauts and IJV thrombosis in at least 1 astronaut, a newly discovered risk associated with spaceflight. Lower body negative pressure may be a promising countermeasure to enhance venous blood flow in the upper body during spaceflight.

## Key Points

**Question:**

Is long-duration exposure to weightlessness associated with impaired cerebral venous outflow and increased risk of jugular venous thrombosis?

**Findings:**

In this cohort study of 11 International Space Station crew members, 6 crew members demonstrated stagnant or retrograde flow in the internal jugular vein on approximate flight day 50, and 1 crew member developed an occlusive internal jugular vein thrombus during spaceflight.

**Meaning:**

Weightlessness is associated with blood flow stasis in the internal jugular vein, which may in turn lead to thrombosis in otherwise healthy astronauts, a newly discovered risk of spaceflight with potentially serious implications.

## Teaser

This cohort study examines the internal jugular vein flow and morphology of crew members of the International Space Station and the use of lower body negative pressure as a countermeasure to the headward fluid shift experienced during space flight.

## Introduction

A gravity-induced head-to-foot (Gz) hydrostatic pressure gradient exists in the fluid-filled systems of the body in the upright position on Earth. In the supine position, the gravity vector no longer pulls in the Gz axis; therefore, blood and tissue-fluid pressures and volumes redistribute across the body. By spending approximately two-thirds of the day upright and the remaining one-third of the day supine at night, humans experience fluid shifts daily. However, crew members on the International Space Station (ISS) are weightless and thus experience a sustained redistribution of fluids toward the head that is not subject to daily diurnal posture-induced change in hydrostatic pressure.^1^ Headward fluid shifts during prolonged weightlessness result in facial puffiness, decreased leg volume, increased stroke volume, and decreased plasma volume.^2,3,4^ This fluid shift may also affect cerebral venous outflow as internal jugular vein (IJV) volume has been showed to be increased during 4.0 to 5.5 months of spaceflight exposure.^5^

The purpose of this study was to quantify the cross-sectional area and pressure of the IJV as well as characterize the Doppler flow velocity profile to describe cerebral venous outflow during spaceflight compared with various postures on Earth. A secondary aim was to evaluate if the use of lower body negative pressure (LBNP) would be associated with negating the effects of the spaceflight-induced headward fluid shift on the IJV.

## Methods

Data were collected as part of the multi-institution international fluid shifts study. All participants provided written informed consent prior to inclusion in the study, and the protocol was reviewed and approved by the National Aeronautics and Space Administration Johnson Space Center institutional review board, internal review boards from additional international space agencies, and the Human Research Multilateral Review Board. This study is reported following the Strengthening the Reporting of Observational Studies in Epidemiology (STROBE) reporting guideline. To protect the identities of the participating ISS crew members, dates for enrollment and data collection are not provided because of attributability issues with the very public nature of our study participants and the paucity of persons in space in a given year.

Potential participants who were scheduled to complete long-duration missions to the ISS were identified, and participants volunteered after receiving a complete description of the study. During preflight and postflight data collection, measurements were acquired in 3 positions: seated, supine, and 15° head-down tilt. Each position was maintained for approximately 45 minutes. Data collection on the ISS occurred without and with 25 mm Hg LBNP. The Russian Chibis-M LBNP used in this study encompasses the lower limbs in a hard enclosure that is sealed at the waist and connected to a vacuum pump to decrease the pressure in the chamber around the lower limbs to subatmospheric pressure.^6^ Lower body negative pressure sequesters fluid volume, mainly venous blood, in the lower extremities and is used by cosmonauts on the ISS as a countermeasure for postflight orthostatic intolerance.^7,8^ Owing to logistical constraints, measurements during LBNP were acquired 10 to 27 days after the first spaceflight data collection session, during which no LBNP was used. During flight, LBNP was applied for approximately 1 hour, with IJV measurements taken approximately 25 minutes after LBNP application. All data collected on the ISS were guided in real time by investigators on the ground.

Cardiac and vascular measurements on the ground and during spaceflight were obtained using the Vivid Q ultrasonographic machine (GE). Stroke volume was calculated as the product of the velocity time integral of the Doppler spectra of the left ventricular outflow tract over 3 cardiac cycles and the cross-sectional area of the aortic root. Heart rate and systolic and diastolic blood pressure were measured using the oscillometric method with an automated brachial blood pressure device on Earth using the Dinamap vital signs monitor (GE) and on the ISS using the Tonoport V ambulatory blood pressure monitor (GE), and mean arterial pressure was calculated. Cardiac output was calculated as the product of heart rate and stroke volume. Cross-sectional images of the left IJV were acquired at end-expiration just below the confluence of the IJV and the superior thyroid vein. The cross-sectional area was manually delineated on 3 images by 2 independent sonographers for all time points. If ultrasonographic measurements for a given image differed by more than 10% between sonographers (20% for seated posture), additional independent sonographers performed the analysis. If the additional analysis did not render a measurement within the prescribed quality-control range, that measurement was not included in the data set.

Pressure within the left IJV was measured using VeinPress compression sonography (Meridian AG) as previously described.^9^ Briefly, the IJV was imaged through a fluid-filled bladder connected to a 12 to 5 MHz linear array ultrasonographic probe. The bladder was attached to a manometer that was zeroed before each measurement. The pressure needed to compress the vein until the walls touched was assumed to equal the pressure within the vein.^9^

**Figure 1.**
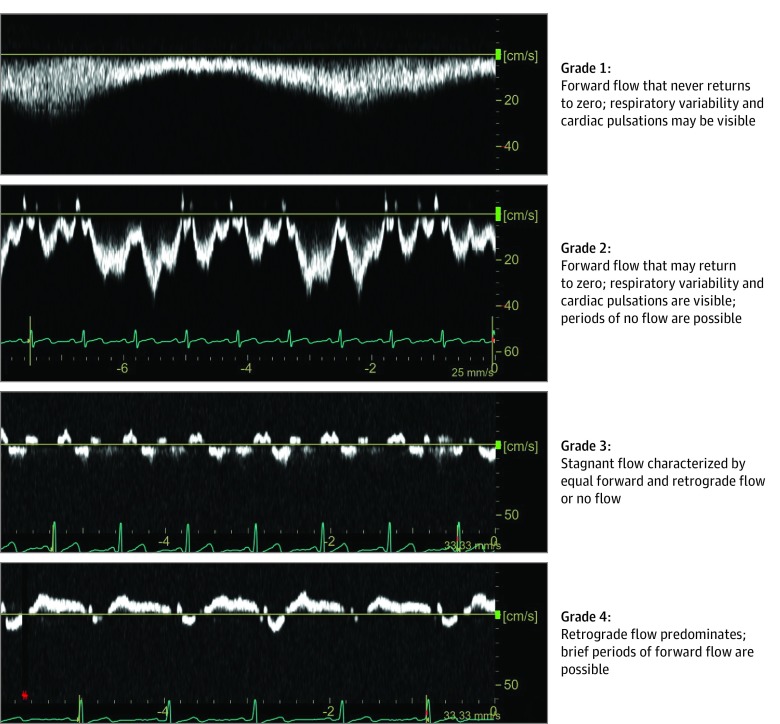
Internal Jugular Vein Blood Flow Velocity Waveform Grading Scale Flow below the baseline (0 cm/s; yellow line) signifies normal flow in the head-to-heart direction, whereas flow above the baseline signifies reverse flow toward the head.

The left IJV blood flow waveform was captured using Doppler ultrasonography with a 12 to 5 MHz linear array probe in the sagittal plane with the sampling window spanning the width of the IJV. Care was taken to collect Doppler data in the same location as the IJV area measurement. The Doppler spectra was acquired during an approximately 5-second period, in triplicate. Owing to cardiac and respiratory variations, the IJV shape and blood flow are dynamic; thus, reporting the velocity at a certain time point may not adequately describe the IJV blood flow status. Therefore, to characterize the venous flow, we developed a 1 to 4 grading system that incorporated both the direction and magnitude of the Doppler signal (Figure 1). Briefly, nominal continuous forward flow (ie, head to heart direction) was scored as grade 1, nominal forward flow with pulsatility was scored as grade 2, stagnant flow was scored as grade 3, and reverse flow toward the head was scored as grade 4. Two trained sonographers independently scored the IJV waveform profiles. Of 101 Doppler images scored, there were 13 initial discrepancies between the 2 sonographers and a consensus was determined by strictly enforcing the grading criteria among the sonography team.

### Statistical Analysis

Statistical analyses were performed using SAS statistical software version 9.4 (SAS Institute) with an emphasis on characterizing the observed effects with modeled means and 95% CIs.^10^ Statistically significant differences were determined against a 2-tailed null hypothesis of no differences with α = 0.05 and 5% false discovery rate corrections for multiple comparisons.^11^ All model assumptions were evaluated prior to reporting effects, resulting in the elimination of a few observations per outcome that produced standardized residuals exceeding 2.5 or −2.5 to meet model assumptions. Our experimental design was longitudinal, with astronauts providing data from each of 3 postures preflight and postflight (seated, supine, head-down tilt), and 2 in-flight conditions observed at 2 periods (spaceflight and spaceflight with LBNP at approximate flight days 50 and 150). We submitted each of our continuously scaled outcomes to separate statistical mixed models, each with a priori fixed-effects parameters comparing preflight and seated with all other postures and times and comparing the spaceflight with LBNP conditions with spaceflight alone at both time points. We included random Y-intercepts to accommodate the within-subject experimental design. *P* values were considered statistically significant at less than .05. Data were analyzed in June 2019.

## Results

Eleven crew members (mean [SD] age, 46.9 [6.3] years; 9 [82%] men; mean [SD] body mass index [calculated as weight in kilograms divided by height in meters squared], 26.4 [3]) participated in the study. The mean (SD) time in space for this cohort was 210 (76) days, and the mean (SD) prior spaceflight exposure was 234 (96) days. Data collection occurred at approximately 95 (range, 53-164) days before launch, on the ISS approximately 50 (range, 33-63) days into spaceflight, with LBNP data collected at 40 to 67 days into spaceflight, and approximately 150 (range 91-161) days into spaceflight, with LBNP data collected a second time at 141 to 181 days, and approximately 40 (range, 32-50) days after return. On Earth, stroke volume and cardiac output increased when going from the seated to the supine position, whereas heart rate decreased (Table). During spaceflight, heart rate and mean arterial pressure were unchanged; however, stroke volume and cardiac output were increased relative to the seated position, but similar to the supine position on Earth. During spaceflight days 50 and 150, application of LBNP, which relocates blood volume to the lower extremities, decreased stroke volume, cardiac output and mean arterial pressure, which were associated with an increase in heart rate (Table).

**Table.** Cardiovascular Measurements Preflight, During Spaceflight, and Postflight

Measurement	Mean (95% CI)									
	Preflight			In-Flight				Postflight		
	Seated	Supine	HDT	Day 50	Day 50 With LBNP^a^	Day 150	Day 150 With LBNP^b^	Seated	Supine	HDT
Stroke volume, mL	69 (63-75)	89 (82-95)	87 (81-93)	86 (80-92)	60 (53-66)	85 (79-92)	57 (50-63)	66 (60-73)	82 (76-89)	91 (84-97)
*P* value	1 [Reference]	<.001	<.001	<.001	<.001	<.001	<.001	NC	NC	NC
Cardiac output, L/min	4.2 (3.7-4.8)	4.8 (4.2-5.4)	4.7 (4.1-5.3)	5.0 (4.4-5.5)	4.2 (3.6-4.8)	5.1 (4.5-5.7)	3.8 (3.3-4.4)	4 (3.4-4.6)	4.8 (4.3-5.4)	5.2 (4.6-5.7)
*P* value	1 [Reference]	<.001	.002	<.001	<.001	<.001	<.001	NC	NC	NC
Heart rate, bpm	61 (58-65)	54 (51-58)	54 (50-57)	60 (57-64)	70 (66-74)	59 (55-62)	7 (68-76)	61 (57-64)	56 (52-60)	56 (52-60)
*P* value	1 [Reference]	<.001	<.001	.61	<.001	.32	<.001	NC	NC	NC
Blood pressure, mm Hg										
Arterial, mean	91 (85-97)	87 (81-93)	88 (82-94)	94 (88-100)	80 (74-86)	90 (84-96)	80 (74-87)	89 (83-95)	85 (79-91)	87 (81-93)
*P* value	1 [Refernece]	.32	.53	.32	<.001	.88	.006	NC	NC	NC
Systolic	119 (112-126)	116 (109-122)	119 (113-126)	123 (116-130)	119 (112-127)	121 (114-127)	116 (108-123)	116 (109-123)	115 (108-122)	119 (112-126)
*P* value	1 [Refernece]	.56	.95	.56	.56	.92	.56			
Diastolic	77 (71-82)	72 (67-78)	73 (67-78)	79 (73-84)	61 (55-66)	75 (69-81)	62 (56-69)	76 (70-82)	69 (64-75)	71 (65-77)
*P* value	1 [Refernece]	.26	.26	.50	<.001	.62	<.001	NC	NC	NC

Abbreviations: bpm, beats per minute; HDT, head-down tilt; LBNP, lower body negative pressure; NC, not calculated.

^a^ *P* values are compared with in-flight day 50 without LBNP.

^b^ *P* values are compared with in-flight day 150 without LBNP.

**Figure 2.**
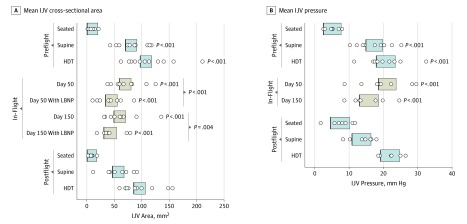
Internal Jugular Vein (IJV) Cross-Sectional Area and Pressure Mean IJV cross-sectional area (A) and pressure (B) were measured in the seated, supine and 15° head-down tilt (HDT) positions preflight, on flight days 50 and 150 of spaceflight, with and without lower body negative pressure (LBNP), and 40 days postflight. Seated preflight measurements were used as the reference. Boxes indicate 95% CIs and circles, individual data points.

During preflight positional changes, mean left IJV cross-sectional area increased from 9.8 (95% CI, −1.2 to 20.7) mm^2^ (ie, partially or fully collapsed) in the seated position to 80.7 (95% CI, 69.9-91.5) mm^2^ in the supine position (*P* < .001) and 107.7 (95% CI, 96.7-118.7) mm^2^ in the head-down tilt position (*P* < .001) (Figure 2A). Compared with preflight seated position, the mean IJV cross-sectional area was greater on flight day 50 (70.3 [95% CI, 59.3-81.2] mm^2^; *P* < .001) and on flight day 150 (60 [95% CI, 48.8-71.2] mm^2^; *P* < .001). Use of LBNP was associated with decreased mean IJV area on flight day 50 (44.7 [95% CI, 33.2-56.2] mm^2^; *P* < .001) and on flight day 150 (42.7 [95% CI, 30.5-54.8] mm^2^; *P* = .004). The postflight response to posture followed a pattern similar to that seen preflight, although the IJV areas during supine and head-down tilt were slightly lower than preflight values (Figure 2A).

Preflight, noninvasive mean IJV pressure responded in a manner similar to the IJV cross-sectional area, increasing from 5.1 (95% CI, 2.5-7.8) mm Hg in the seated position to 17.3 (95% CI, 14.8-19.8) mm Hg in the supine position (*P* < .001) and to 20.7 (95% CI, 17.8-23.5) mm Hg in the head-down tilt position (*P* < .001) (Figure 2B). During spaceflight, the mean IJV pressure was higher than seated preflight values on flight day 50 (21.1 [95% CI, 18.5-23.7] mm Hg; *P* < .001) and flight day 150 (15.8 [95% CI, 13-18.6] mm Hg; *P* < .001), and there was a decrease from day 50 to day 150 on the ISS. After return to Earth, mean IJV pressure responded to positional changes in the same manner as before flight.

**Figure 3.**
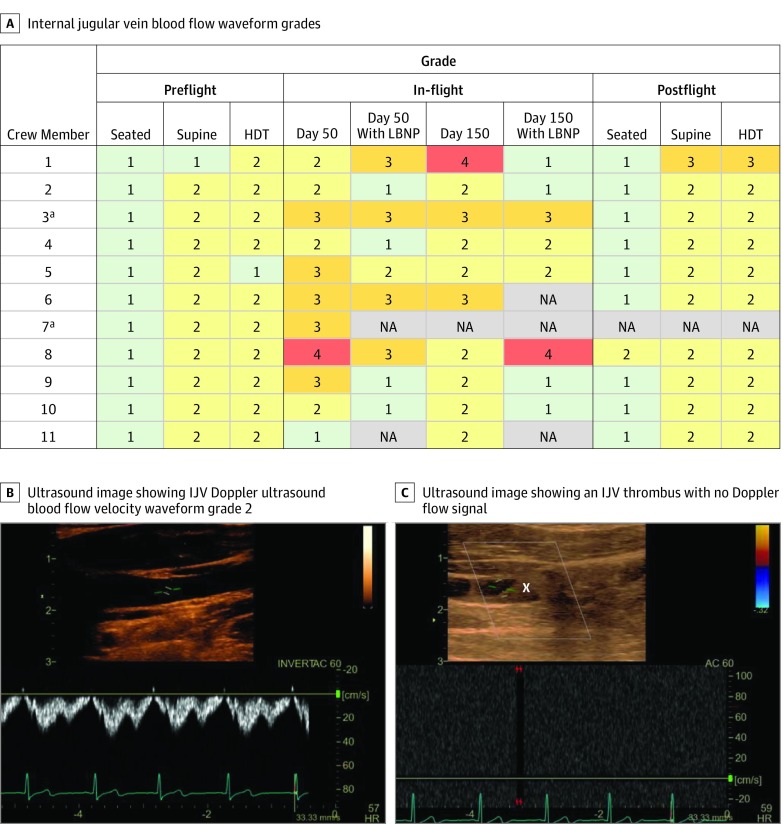
Internal Jugular Vein (IJV) Blood Flow Waveform Grading Before, During, and After Spaceflight and Example Ultrasonography Demonstrating the Presence of a Thrombus During Spaceflight Grade 1 indicates predominantly continuous flow; grade 2, predominantly pulsatile flow that may return to 0; grade 3, no net forward flow (stagnant or equal positive and negative flow); grade 4, retrograde flow. HDT indicates 15° head-down tilt and LBNP, lower body negative pressure (A). Example longitudinal ultrasonographic scans of the left IJV and Doppler ultrasonographic blood flow velocity waveform in one participant showing grade 2 flow preflight measured in the supine position (B), and a thrombus (denoted with an X) with no flow detected through the IJV on flight day 50 on the International Space Station (C). ^a^Denotes crew members who presented with an IJV thrombus during spaceflight.

The scoring system developed to describe changes in the left IJV blood flow waveform revealed changes consistent with posture on Earth. Preflight, all crew members demonstrated grade 1 flow in the IJV in the seated position with predominantly continuous waterfall-like flow. During supine and head down tilt, 10 of 11crew members (91%) demonstrated grade 2 pulsatile flow with no evidence of stagnant or reverse flow (Figure 3A). During spaceflight, the left IJV blood flow was altered relative to all preflight postures with stagnant blood flow (grade 3, Video 1; eFigure 1 in the Supplement) in 5 of 11 crew members (45%) on flight day 50 and 2 of 10 crew members (20%) on flight day 150. Furthermore, 1 crew member presented with a predominately retrograde flow pattern on flight day 50 (grade 4, Video 2; eFigure 2 in the Supplement), and another crew member developed retrograde flow on flight day 150. Of 10 crew members with data from flight day 50 to flight day 150, 5 (50%) had no change in IJV flow grade, 3 (30%) had improved flow, and 2 (20%) demonstrated diminished flow (Figure 3). Nine crew members participated in LBNP application on flight day 50, and 8 crew members participated on flight day 150. Of the 17 LBNP sessions during spaceflight, 10 sessions (59%) were associated with improved IJV blood flow (lower grade), whereas 2 sessions were associated with worsened flow (higher grade), and 5 sessions (29%) were not associated with any change to IJV blood flow.

**Video 1.** Stagnant Internal Jugular Vein Blood Flow During Spaceflight Blood in the internal jugular vein can be seen moving back and forth with no net forward flow toward the heart (to the right), demonstrating stagnant (grade 3) flow.

**Video 2.** Retrograde Internal Jugular Vein Blood Flow During Spaceflight Blood in the internal jugular vein is flowing in the reverse direction (toward the head, left) demonstrating retrograde (grade 4) flow.

Notably, of the seven crew members who demonstrated stagnant or retrograde IJV blood flow during spaceflight (grade 3 or 4), 1 crew member developed an occlusive thrombus that was discovered on flight day 50 (Figure 3B) and confirmed with a follow-up clinical ultrasonographic assessment of compressibility and response to respiratory maneuvers. Diagnosis was based on review of the examination by 2 independent radiologists with extensive experience in thrombus detection and diagnosis. The crew member was subsequently treated with anticoagulants for the remaining duration of the flight and did not participate in further data collection for this study past flight day 50. After this incidental finding, a retrospective review of all IJV ultrasonographic data was performed by a multi-institutional panel of experts. A heterogeneous mass identified as a partially occlusive thrombus was discovered in the left IJV of a second crew member, however, no compression was possible in this case owing to the retrospective nature of the finding.

## Discussion

In this cohort study, we evaluated the IJV structure and function associated with acute headward fluid shifts during postural changes on Earth and compared these values with those observed during long-duration spaceflight when participants were exposed to a sustained headward blood and tissue fluid shift. To our knowledge, this is the first study to demonstrate blood flow stasis in the left IJV in approximately half of crew members during spaceflight and jugular venous thrombosis in some crew members, a previously unrecognized risk of spaceflight. Although IJV blood flow was altered during spaceflight, IJV area and pressure were similar to supine values on Earth. Notably, LBNP was associated with reduced IJV area and improved blood flow patterns in most LBNP sessions during spaceflight and thus may be a promising countermeasure to blood flow stasis and thrombosis associated with spaceflight.

Cerebral venous outflow occurs predominantly through the IJVs in the supine position, whereas in the upright position, the IJVs partially or fully collapse due to atmospheric pressure being greater than intraluminal pressure, and cerebral venous outflow is diverted to the vertebral veins and vertebral plexus.^12,13^ However, in most cases, the IJVs do not completely occlude in the upright posture, and fluid communication is present between the cerebral and central venous systems.^12,14^ Indeed, we observed a waterfall-like blood flow pattern through the left IJV in most participants during upright posture on Earth, despite the semicollapsed state of the vein. Cerebral venous drainage plays an important role in regulating intracranial pressure and intracranial fluid dynamics,^15^ and changes in cerebral venous pressure result in corresponding changes in intracranial pressure.^14,16^ In the upright position, venous pressure along the Gz body axis changes with respect to the venous hydrostatic indifference point because of hydrostatic effects. Notably, by collapsing, the IJVs act as Starling resistors, a protective mechanism preventing severely negative intracranial pressure in the upright position. The IJV cross-sectional area and flow are modulated by both cardiac and respiratory cycles and can be influenced by posture, anatomical variations, incompetence of the jugular valve, and changes in central venous or intrathoracic pressure. Pressure in the IJV is increased during short periods of weightlessness in parabolic flight,^9^ and in this study, we found that the IJV pressure remained elevated during long-duration spaceflight on the ISS relative to the upright posture. This result concurs with previously measured increases in intracranial pressure and transmural central venous pressure during microgravity exposure relative to the upright position on Earth.^17,18^ Our results also expand on previous reports of engorged IJVs during short- and long-duration spaceflight^5,19^ and frame the extent of the engorgement by comparing the IJV area to 3 postures on Earth; however, it is possible that cross-sectional area measures underestimated the extent of engorgement compared to volume measures of the IJV.^5^ Cephalad fluid shifts, secondary to the lack of gravitational vectors and the reduction of tissue weight and tissue compressive forces in microgravity, may account for the increase in IJV area and pressure.

We found that in microgravity, crew members were exposed to constant cerebral venous congestion with the potential to develop stagnant venous blood flow. The triad of Virchow describes the 3 main factors that contribute to thrombosis as stasis of flow, hypercoagulability, and endothelial injury or dysfunction. Notably, stasis of blood flow can lead to local elevations of various hemostasis-activation factors, increase blood cell–endothelium interaction, and create local hypoxia-induced endothelial activation, all factors that can lead to thrombosis.^20^ Thus, sustained stagnation of blood flow in the IJV during spaceflight may create an increased risk for thrombus formation. Risk factors associated with venous thrombosis on Earth include recent surgery, trauma, age, cancer, thrombophilia, catheter insertion, infection, pregnancy or puerperium, and use of oral contraceptives.^20,21,22,23^ As all astronauts undergo comprehensive medical screenings and are considered healthy individuals, many venous thrombosis risk factors are not applicable to the spacefaring population, with the exception of possible oral contraceptive use. Estrogen-containing contraceptives have received extensive use in human spaceflight for menstrual suppression. The increased risk of venous thrombosis with use of oral contraceptives is well established,^24,25,26,27^ and combined with weightlessness-induced blood flow stasis in the IJV during spaceflight, may lead to increased risk for development of thrombosis. However, it should be noted that the incidences of IJV thrombi in this study developed in both female and male crew members. In addition, astronaut candidates are not systematically screened for thrombophilia, such as antithrombin, protein C, and protein S deficiencies.^20^ Anatomical IJV abnormalities, including stenosis, hypoplasia, and abnormal flow, may also play a role in thrombosis formation.^28,29,30^

Humans have been flying in space for more than 50 years, yet this is the first report of venous thrombosis during spaceflight, to our knowledge. Given that the thrombi detected in our study were asymptomatic and only discovered in the course of assessing the IJV, it is plausible that undetected thrombi have occurred previously during human spaceflight missions, albeit without negative clinical outcomes attributed to thrombi sequelae to date.

To counteract the headward fluid shift in space and improve blood flow patterns, LBNP was tested as a countermeasure, drawing venous blood away from the head and into the lower extremities. During spaceflight, LBNP was associated with reducing IJV area, but the reduction did not reach seated baseline IJV values. In addition, the left IJV blood flow waveform pattern improved in 59% of LBNP sessions during spaceflight. Notably, 3 crew members had their flow pattern improve from stagnant or reverse (grade 3 or 4) to nominal flow (grade 1 or 2). This suggests that LBNP may be able to acutely improve IJV flow and could potentially reduce thrombosis risk; however, optimal LBNP exposure time and frequency are unknown. Furthermore, there are potential risks associated with LBNP, including possibility of syncope during application; thus, medical monitoring is warranted. Further research is needed to determine if there are any sustained beneficial vascular effects after LBNP application as well.

### Limitations

This study had several limitations. First, as it was a prospective research study, we are unable to comment on further medical treatments of the thrombus. Second, as the IJV does not have a hard surface (ie, bone) directly behind it to compress against, the noninvasive pressure device used in this study likely overestimated the absolute pressure of the IJV; however, the results were useful in comparing relative changes. Third, only the left IJV was imaged as part of this study; thus, we are unable to comment on the presence or absence of blood flow stasis in the right IJV. However, the right IJV has been examined previously during spaceflight with no reported signs of blood stagnation or thrombosis.^5,19^ Thus, anatomical differences and the nondominant nature of the left IJV in most healthy individuals may have contributed to blood stagnation in the left IJV during spaceflight.

## Conclusions

This cohort study’s findings of abnormal and stagnant cerebral venous outflow in the IJV during spaceflight and subsequent development of jugular vein thrombosis are novel findings that may have significant human health implications for civilian spaceflight as well as future exploration-class missions, such as a mission to Mars. The potential relationship between altered cerebral venous outflow and the spaceflight associated neuro-ocular syndrome^31^ and neurocognitive performance should be further investigated. Our findings highlight the need for a more comprehensive evaluation of bilateral venous hemodynamics during spaceflight, as well as for investigation of countermeasures, including LBNP, that can restore vascular physiology to a state similar to that seen in the upright and supine positions on Earth.

## Notes

Supplement. **eFigure 1.** Doppler Ultrasonographic Tracing of the Internal Jugular Vein With Stagnant Blood Flow **eFigure 2.** Doppler Ultrasonographic Tracing of the Internal Jugular Vein With Reverse Blood Flow
