# Error in Short Title and Table

**DOI:** 10.1001/jamanetworkopen.2019.20195

**Published:** 2020-01-03

In the Original Investigation titled “Assessment of Jugular Venous Blood Flow Stasis and Thrombosis During Spaceflight,”^1^ published November 13, 2019, there were typographical errors in the short title, which should have read, “Assessment of Jugular Venous Blood Flow Stasis and Thrombosis During Spaceflight,” and in the Table: mean heart rate at in-flight day 150 with lower body negative pressure should have read “72,” and preflight seated *P* values should have read “Not applicable.” This article has been corrected.
