# Supplementary material for: Assessment of Jugular Venous Blood Flow Stasis and Thrombosis During Spaceflight
Source: JAMA Netw Open. 2019 Nov 13;2(11):e1915011. doi: 10.1001/jamanetworkopen.2019.15011 (PMC6902784; doi:10.1001/jamanetworkopen.2019.15011)
Supplement: Supplement. — eFigure 1. Doppler Ultrasonographic Tracing of the Internal Jugular Vein With Stagnant Blood Flow eFigure 2. Doppler Ultrasonographic Tracing of the Internal Jugular Vein With Reverse Blood Flow [file jamanetwopen-2-e1915011-s001.pdf]

Supplementary Online Content

Marshall-Goebel K, Laurie SS, Alferova IV, et al. Assessment of jugular venous blood flow stasis and thrombosis during spaceflight. *JAMA Netw Open*. 2019;2(11):e1915011. doi:10.1001/jamanetworkopen.2019.15011

eFigure 1. Doppler Ultrasonographic Tracing of the Internal Jugular Vein With Stagnant Blood Flow

eFigure 2. Doppler Ultrasonographic Tracing of the Internal Jugular Vein With Reverse Blood Flow

This supplementary material has been provided by the authors to give readers additional information about their work.

© 2019 Marshall-Goebel K et al. *JAMA Network Open.*

eFigure 1. Doppler Ultrasonographic Tracing of the Internal Jugular Vein With Stagnant Blood Flow

Legend: With the sample volume taken in the internal jugular vein lumen (A), the resulting Doppler tracing of blood flow in the internal jugular vein (B) during spaceflight shows predominately stagnant flow (grade 3), with no net forward flow towards the heart as would nominally be seen in the vessel on Earth.

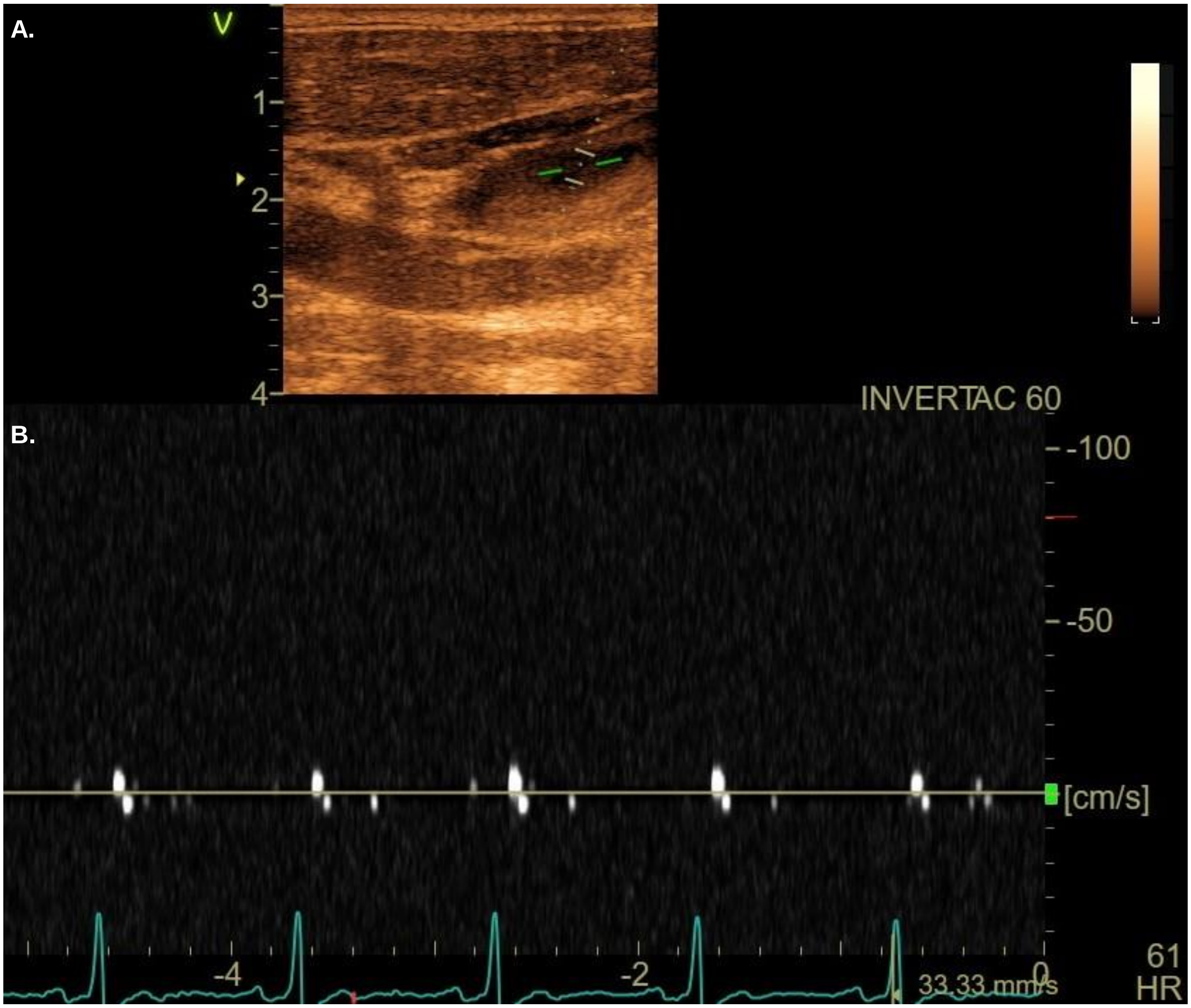

© 2019 Marshall-Goebel K et al. *JAMA Network Open.*

eFigure 2. Doppler Ultrasonographic Tracing of the Internal Jugular Vein With Reverse Blood Flow

Legend: With the sample volume taken in the internal jugular vein lumen (A), the resulting Doppler tracing of blood flow in the internal jugular vein (B) during spaceflight shows predominately retrograde flow towards the head (grade 4), the opposite of what would normally be seen on Earth.

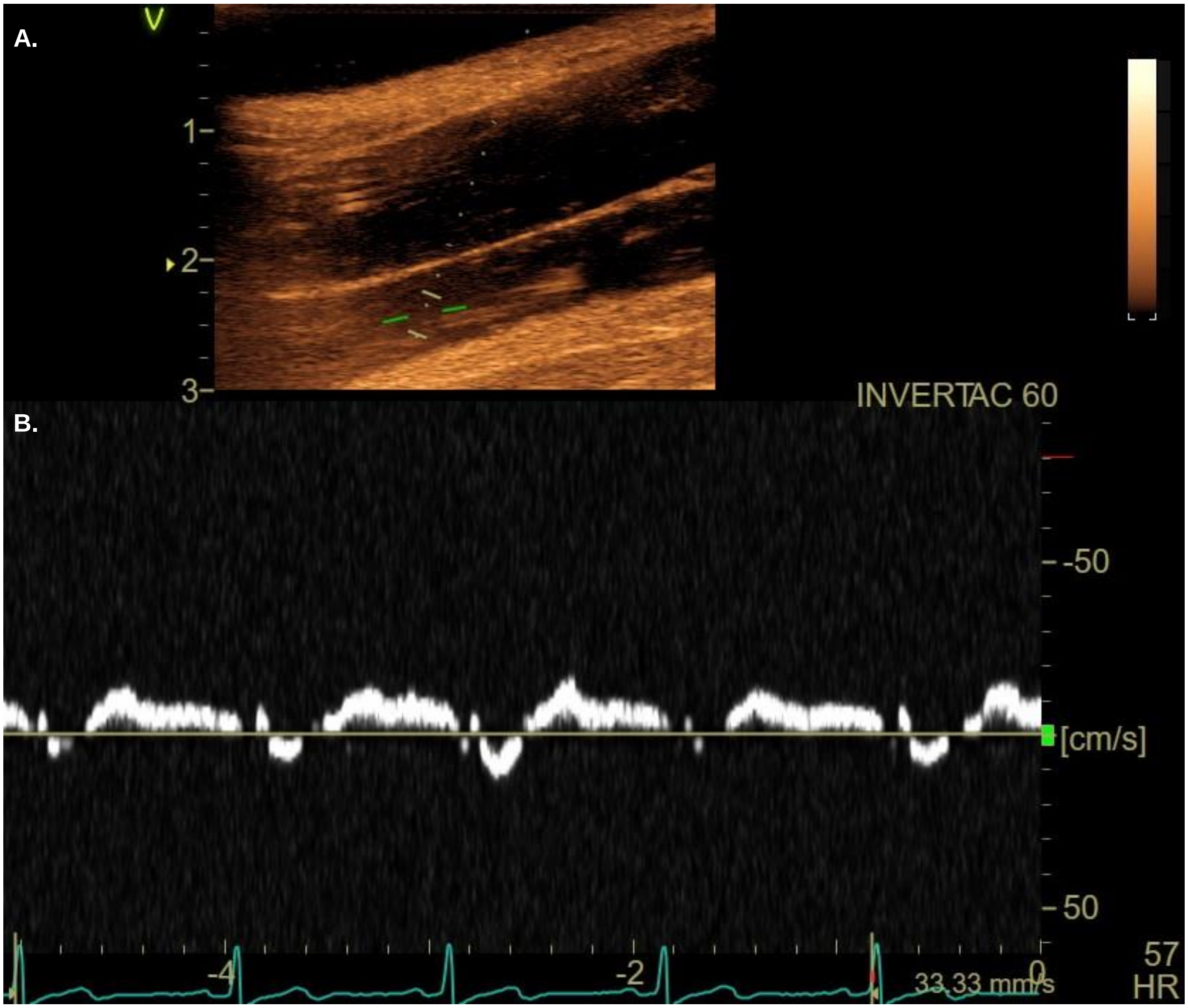

© 2019 Marshall-Goebel K et al. *JAMA Network Open.*
